# The influence of tip shape on bending force during needle insertion

**DOI:** 10.1038/srep40477

**Published:** 2017-01-11

**Authors:** Nick J. van de Berg, Tonke L. de Jong, Dennis J. van Gerwen, Jenny Dankelman, John J. van den Dobbelsteen

**Affiliations:** 1 Delft University of Technology, BioMechanical Engineering, Delft, 2628CD, The Netherlands

## Abstract

Steering of needles involves the planning and timely modifying of instrument-tissue force interactions to allow for controlled deflections during the insertion in tissue. In this work, the effect of tip shape on these forces was studied using 10 mm diameter needle tips. Six different tips were selected, including beveled and conical versions, with or without pre-bend or pre-curve. A six-degree-of-freedom force/torque sensor measured the loads during indentations in tissue simulants. The increased insertion (axial) and bending (radial) forces with insertion depth — the force-displacement slopes — were analyzed. Results showed that the ratio between radial and axial forces was not always proportional. This means that the tip load does not have a constant orientation, as is often assumed in mechanics-based steering models. For all tip types, the tip-load assumed a more radial orientation with increased axial load. This effect was larger for straight tips than for pre-bent or pre-curved tips. In addition, the force-displacement slopes were consistently higher for (1) increased tip angles, and for (2) beveled tips compared to conical tips. Needles with a bent or curved tip allow for an increased bending force and a decreased variability of the tip load vector orientation.

The occurrence of needle deflections in clinical practice is typically attributed to unbalanced force interactions at the tip. This imbalance occurs during the insertion phase and results from the needle itself, which may be asymmetric in shape, or from uneven properties or boundary conditions within the tissue.

Despite the limited theoretical knowledge on the formation of needle-tissue contact loads, the utility of bending forces for the purpose of needle steering has been explored at lengths over the past decades^1^. Often, needle shape estimates rely on mechanical models that describe the movement constraints of a deflecting rod, suspended and propagating within a soft, elastic material^2^. For top-down — descriptive — models that summarize the needle kinematics, tip-tissue interactions do not have to be understood in detail. One can directly relate an input variable, e.g. the duty cycle of a rotating bevel-tip needle, to an output metric, e.g. the radius of curvature of the resulting needle path^3^. For bottom-up — explanatory — models, the underlying causes of observed deformations and loading conditions are relevant. For example, one can estimate the tip force by means of a force orientation prediction, e.g. orthogonal to a bevel surface, an axial insertion force measurement, and a friction estimate obtained from test sets^4^. Current mechanics-based models distinguish the forces at play, but do not yet provide insight into their constitution. It is not yet known how the insertion method, the tissue environment, and the tip geometry affect the mechanics, and neither is it known how this type of information should be processed in navigation models.

To arrive at parametric relations of the asymmetric tip-tissue interactions, supportive force-displacement data are needed. An analysis of deflections of needles with various tips has been provided by Sitzman and Uncles^5^. Okamura *et al*. measured insertion forces in silicone at the base of bevel, conical, and triangular tipped needles^6^. By means of a radial-to-axial force ratio, they showed that bevel-tip needles bend more than conical or triangular-tip needles. They also noted that axial force may decrease with the number of sharp edges at the tip. Podder *et al*. measured the six-degree-of-freedom (6-DOF) force-torque response during *in-vivo* prostate brachytherapy interventions, using diamond-tip needles^7^. The maximum insertion force was on average 15 N, compared to a bending force of approx. 1 N for these symmetric tips. Wedlick *et al*. studied curvature for various arc lengths of pre-curved needles in plastisol^8^. They showed that the radius of curvature of the tip path was inversely proportional to the arc length and also that steering of pre-curved needles was dependent on the insertion velocity. By means of a novel macroscopic approach, Misra *et al*. revealed that the transverse forces at the needle tip decreased with increased bevel angles^9^. Needles, 15 mm in diameter, with bevel angles between 10° and 60°, were inserted in plastisol. Transverse forces up to 4.4 N were measured, but corresponding insertion forces were not reported. The presented parametric tip force and tip moment relations were considered to be independent of the input force. Majewicz *et al*. studied the needle curvature in *ex-vivo* goat liver^10^. They compared multiple bevel angles, but could not conclude that there was a difference in curvature. They suggested this was a result of the increased tissue inhomogeneity and viscoelasticity of biological tissue, compared to artificial tissue. An increase in needle curvature with pre-bent angle was found. In a continuation of this work, insertion forces were measured during puncturing of canine prostate, kidney, and liver tissue using conical, beveled, and pre-bent beveled tips. Tip type did not have a clear effect on the required insertion force, but forces did differ among the tested organs^11^.

The above work reveals that descriptive modeling of bending mechanics in real tissue can be complex. For the development of explanatory models, it is useful not only to study the system as a whole, but also to focus on isolated parts of the system. For instance, studies have analyzed the fracture mechanics at the tip^12^ and the friction along the shaft^13^. Another factor, which is expected to be crucial in the constitution of an asymmetric tip load and in the deflection of a needle, is the tip-tissue contact force. With the exception of beveled needles^9^, this factor has only been discussed implicitly as a contributing factor to the complete-system mechanics.

The present study aims to provide a quantitative comparison of tip-tissue contact forces, measured with a 6-DOF force-torque (F/T) sensor, during the insertion of various needle tips in simulant tissue. The insertion (axial) force, the bending (radial) force, the radial-to-axial force ratio, and the slopes of the force-displacement curves were studied in relation to the tip geometry by minimizing obscuring factors, such as non-linear tissue elasticity, friction, and fracture forces. This required the use of homogeneous tissue simulants. The influence of the choice of tissue simulant was assessed by performing experiments in both silicone-based and agar-agar-based materials. The conducted study is relevant for the formulation of explanatory, mechanics-based models, the optimization of tip designs, and the proper actuation of shape-adaptable needles, such as concentric tubes^14^, compliant needles^15^, and articulated-tip needles^16^,^17^,^18^.

## Materials and Methods

### Measurement setup

**Figure 1:**
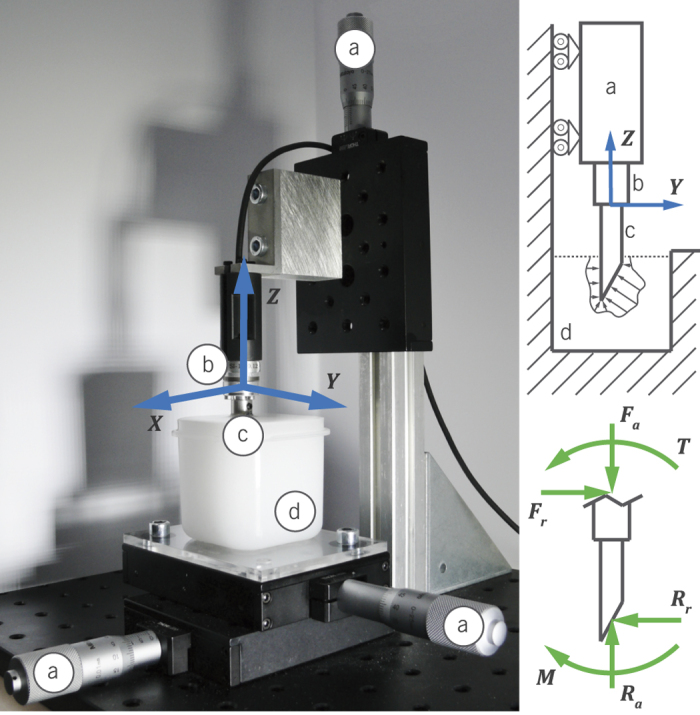
Experimental setup showing (**a**) the manual stages, (**b**) the force sensor with global coordinate system, (**c**) the needle tip, and (**d**) the tissue phantom. On the right, a schematic mechanical representation of the measurement is shown, including the axial and radial forces, *R*_*a*_ and *R*_*r*_, and the resultant moment *M*, at the tip.

The experimental setup consisted of a manual *XYZ*-translation stage built up from three single axis micro-positioning stages with a 10 *μ*m resolution (PT1/M, Thorlabs, US). A container of size 55 × 55 × 55 mm was glued onto a PMMA plate and fixed to the *XY*-stage platform, as shown in Fig. 1. During the experiments, the container was filled with artificial tissue. The *XY*-platform allowed for a maximization of the side-wall clearance for the various tip shapes. The *Z*-stage was used to insert the tips and thereby alter the magnitude of the tip-tissue loads. As a result of the sensor-tip alignment, *F*_*z*_, *F*_*y*_ and *T*_*x*_ were expected to be the dominant force and torque components in the experiment. Their relations to the insertion loads (*F*_*a*_, *F*_*r*_, *T*) and tip-tissue contact loads (*R*_*a*_, *R*_*r*_, *M*) are defined in the *Data processing* section. A 6-DOF *F*/*T* sensor (ATI nano17, ATI Industrial Automation, US), was used to collect the loads exerted at the base of the needle tip. This sensor was connected to an amplifier (BPS4000, Calex Electronics Limited, GB), and a 16-bit DAQ system (NI USB-6210, National Instruments Corporation, US). Force data were sampled at 200 Hz.

### Needle tips

**Figure 2:**
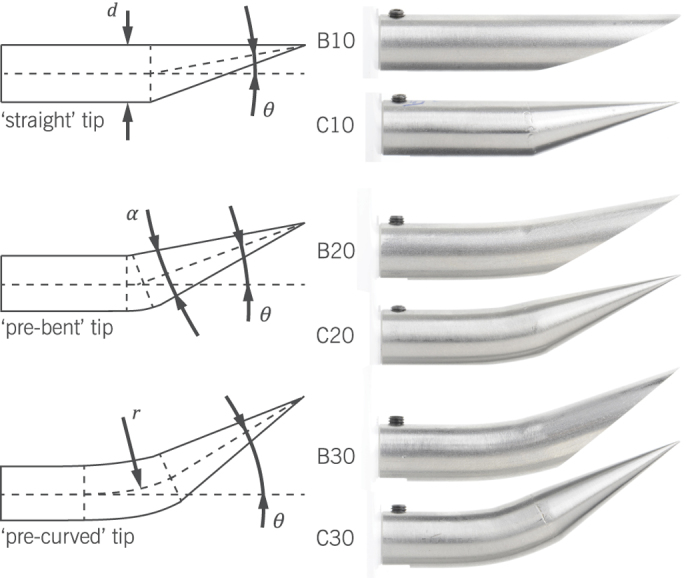
Overview of the needle tips produced for this study. Shown on the left are the side-view projections of the tips, with or without pre-bend or pre-curve. On the right, photographs of the actual needle tips are shown. The tip types are denoted by a B or C, corresponding to a bevel or conical tip, and a number corresponding to the tip angle, *θ*, which was either 10°, 20°, or 30°. In the side-view projection, all six tips had an equal apex angle, *α* = 20°. The needle diameters were also equal, *d* = 10 mm, and the radius of curvature was *r* = 40 mm.

The needle tips used in this study were scaled-up models with an outer diameter (*d*) of 10 mm, allowing a similar macroscopic approach as in ref. 9. Based on previous work on shape-adaptable needles^15^,^16^,^17^,^18^, six different tip shapes were selected and compared. This is a subset of the tip types presented in the overview of van Gerwen *et al*.^19^. This includes three bevel types and three conical types, each with a straight, pre-bent or pre-curved configuration, shown in Fig. 2. These tips were chosen in such a way that they formed complementary pairs in terms of their side-view surface projection. In this projection, each tip had an apex angle (*α*) of 20°. Note that straight (symmetric) conical tips were not used. Instead, two versions of pre-bent conical tips, with tip angles (*θ*), of respectively 10° and 20°, were used. The latter corresponds to the actuation limit of the articulated-tip needle described in ref. 17. In analogy, the pre-curved tips correspond to steerable needles with a compliant joint near the tip, where *θ* = 30°^16^. The six tip types were denoted by a B or C for a beveled or conical tip, followed by a number that matched the tip angle. For the sake of similarity, the tip angle was defined by the line connecting the needle centerline to the sharp end at the tip, shown by the dotted lines in Fig. 2. For the straight beveled tip this meant that the tip angle equaled half the bevel angle.

### Specimens

Since fracture phenomena are difficult to control, they were avoided by (1) embedding the needles in tissue prior to testing, and (2) applying only small tip displacements. Two tissue simulants were selected that respectively cured and congealed at room temperature: silicone rubber and agar-agar. After the tissue simulant preparation, one of the tips was fixed to the force sensor and lowered in the container. The tip was embedded in the simulant material with an initial depth of 45 mm. In total, for each simulant materials, thirty containers were prepared using the protocols described below. The specimen hardness was estimated in simulant tissue test-sets, using a Shore OO durometer (HT-6510-OO, Landtek, CN), with a 2.4 mm diameter spherical indenter (18 mm in width).

#### Silicone preparation

The silicone material (Dragon Skin 10 Medium, Smooth-on, US) consisted of two components (*A* and *B*) mixed at a 1:1 ratio. The specified hardness for this material was 10 Shore A. Based on a proposed tissue simulant for needle insertions by Wang *et al*.^20^, the silicone was mixed with a 40 wt.% paraffin-based oil (baby oil, Johnson & Johnson, US). This reduced the material stiffness and the friction between the tip and its environment^20^. For each container, 60 g oil was mixed with 45 g silicone component *A*. After stirring, 45 g silicone component *B* was added. The mixture was again stirred and poured in the container. The silicone was left to cure with the appropriate needle tip in place for at least five hours. No vacuum chamber was used for the silicone preparation. After curing (5 h), the average material hardness over ten indentations was 33.8 ± 2.5 Shore OO.

#### Agar-agar preparation

Since organic, gelatin-like phantoms are frequently used in needle insertion studies, a comparison study was conducted in a 2.5 wt.% agar-agar in water solution (Agar Agar, Pit & Pit, BE). For this, 4 g of agar-agar powder was mixed with 156 g water and heated in a water bath under constant stirring, until the turbid suspension became a clear solution. This typically occurred at around 84 °C. To reduce heat conduction to the force sensor, the assembly was delayed until the agar-agar solution cooled to 65 °C. Further congealing occurred at room temperature, with the needle in place, over a time span of at least six hours. Due to a limited material elasticity, accurate hardness measurements could not be obtained. Shore hardness measurements started at approx. 50 Shore OO, but quickly dropped below 20 Shore OO, as the durometer probe plastically deformed the material. For a detailed evaluation of mechanical properties of agar-agar, the reader is referred to ref. 21. A low friction between tip and phantom was observed, which likely resulted from the high water content of agar-agar.

### Experimental design and protocol

Tip shape (B10, B20, B30, C10, C20, C30) and specimen material (silicone, agar-agar) were selected as independent variables, and the force resultant measured at the base of the needle tip was the primary dependent variable. For practical reasons, a separate experiment was conducted for each specimen material. For both experiments, thirty specimens were prepared in total, in a sequential order. The six tip shapes were assigned to these specimens in a random order, with the restriction that each tip shape must occur five times.

During the experiments, the force sensor and manual stages were kept fixed to the frame and only the tips were replaced. A pin-in-hole tip-sensor connection with a set screw ensured a steady tip-stage alignment. A slight spread in the axial tip orientation could occur, which was measured by the force component orthogonal to the theoretical symmetry plane (*F*_*x*_).

A single specimen was subjected to *n* load cycles (*n* = 5 for silicon rubber, *n* = 1 for agar-agar, due to a limited elastic material recovery in agar-agar). One load cycle consisted of stepwise changes in needle position from *z*_*ref*_ to *z*_1*N*_, *z*_2*N*_, and *z*_3*N*_, using the manual positioning stage. Here, *z*_*ref*_ was the initial position, which was constant for each run and corresponded to an unknown axial force. It was unknown in the sense that the pre-load was somewhat variable, e.g. due to curing or congealing processes. The position *z*_1*N*_ corresponded to an increase in axial force of 1 N with respect to *z*_*ref*_, and so on. The force and torque histories corresponding to each step were recorded into separate files on a computer, and the translations required to reach the desired axial forces (e.g. *z*_1*N*_) were noted down.

### Data processing

**Figure 3:**
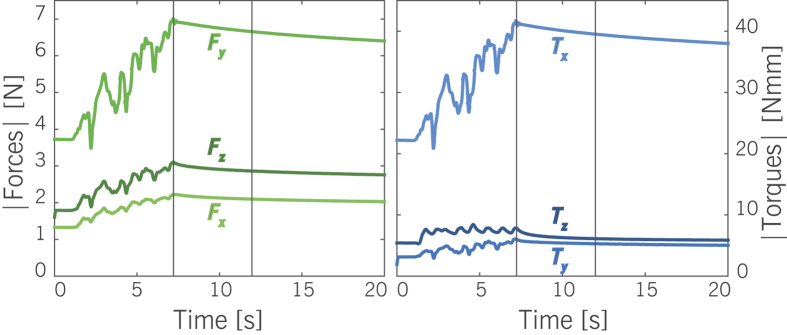
Filtered data from a typical measurement in agar-agar. Forces (left) and torques (right) increased during manual setting of *F*_*z*_ (here to 3 N), and dropped gradually once the insertion stroke was paused. The measurement interval is visualized by two black vertical lines.

The measured *F*/*T* data were processed with a zero-phase moving average filter, using a kernel size of twenty. Figure 3 shows a typical example of filtered data in the force sensor coordinate system (the global *XYZ*-frame), for one of the measurements in agar-agar. The absolute *F*/*T* values are shown to allow for a straightforward comparison of magnitudes. The actual directions or signs are visible from Fig. 1. As similar effects were seen in both the force and torque response, the results section is primarily focused on the force data.

As the tips in this study have a symmetry plane, a force balance can ideally be solved in this plane (see Fig. 1). Measured *F*_*x*_ values could have resulted from either nuisance or from variability in the axial orientation of the tip. To account for this, a radial force, *F*_*r*_, was defined by vector addition of *F*_*y*_ and *F*_*x*_. This equals rotating of the global frame, with an angle *ϕ*, to a local frame that is aligned with the tip’s symmetry plane. The local coordinate frame is denoted in 2-D by *F*_*r*_ and *F*_*a*_, where *r* stands for the radial, and *a* for the axial force component. In this transformation, *F*_*a*_ equals *F*_*z*_. Subsequently, it is assumed that the axial and radial contact forces at the tip, *R*_*a*_ and *R*_*r*_, can be approximated by the measured loads at the base, so that *R*_*r*_ = *F*_*r*_, and *R*_*a*_ = *F*_*a*_.

Figure 3 shows that *F*/*T* magnitudes gradually decay with time after reaching a loading step. Measurement intervals were defined as the first 5 s upon reaching a new loading condition. This event was identified in the *F*_*z*_ data by means of a peak search function in MATLAB. Over each interval, the radial-to-axial force ratio (*F*_*r*_/*F*_*a*_) was determined, in a similar approach to Okamura *et al*.^6^. Note that this equates to finding the tangent of the tip load orientation in the *a*-*r*-plane. It was found that the decay rates of force components were similar, and that this force ratio was nearly constant for most of the measurements. Expressed as a percentage of the initial value[mean, max], the force ratio decrease over the measurement interval was [2%, 11%] in agar-agar, and [2%, 15%] in silicone. To account for the few measurements that remained skewed, the median forces per interval were used as the data points.

Finally, the force-displacement relations in silicone were analyzed by means of simple linear regressions. Linear relations were assumed for small insertion depths, whereas the overall force-displacement curves may have been non-linear. As current mechanics-based system models often split-up the axial and radial force components, individual slopes for these components were provided. A coefficient of determination (*R*^2^) showed how much of the measurement variability could be explained by these linear models.

## Results

### Tip-load orientations

**Figure 4:**
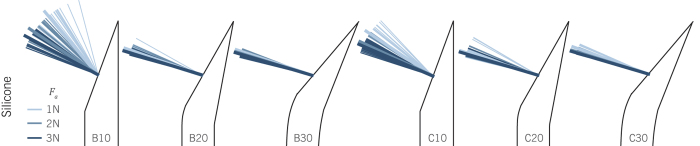
Summary of resultant force vector orientations per tip type and axial loading condition in silicone. The slightly thicker and longer lines present the median vector orientations per experimental condition.

Figure 4 shows the orientation of the measured force resultants per applied insertion (axial) force condition (*F*_*a*_ = 1, 2, and 3 N), during the experiment in silicone. The figure does not contain information on force magnitudes. For an easy comparison, all tip-load vector origins are drawn at the center of the tip-tissue contact surface. In reality, origin locations may deviate. Vectors are color-sorted with respect to *F*_*a*_. The median tip-load orientation, for each tip type and loading condition, is highlighted by means of a slightly thicker and longer line. The order in which these median observations occurred was equal for all six tip types, suggesting that an increased insertion force caused a more radial orientation of the tip load. The tip-load orientation data for the agar-agar experiment can be found as Supplementary Fig. S1.

### Tip-load magnitudes

**Figure 5:**
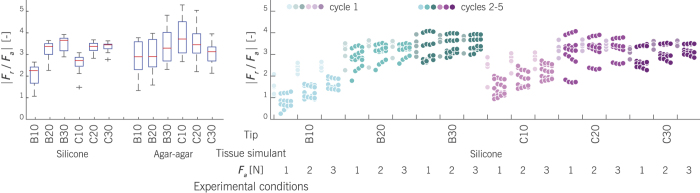
Data summary of the bending force per 1 N of insertion force (radial-to-axial force ratio) for various beveled and conical tip types, measured in two phantom materials. The left figure shows box plots of the first loading cycle in silicone and agar-agar tissue simulants (*n* = 15 per box). The right figure illustrates how this metric changes with subsequent loading cycles in silicone. During a loading cycle, tip loads were measured at the axial displacements *z*_*ref*_, *z*_1*N*_, *z*_2*N*_, and *z*_3*N*_, where *ref* denotes the reference condition and the other subscripts denote the applied axial force, *F*_*a*_.

Bending force magnitudes are expressed per 1 N of insertion force by means of a radial-to-axial force ratio (*F*_*r*_/*F*_*a*_). Figure 5 shows a summary plot of all the collected data, where the force ratio is sorted by phantom tissue, tip type, and applied axial force. The left hand side of the figure compares the force response during the first loading cycle in silicone and agar-agar phantoms. Between simulants, the largest differences in the force ratio location were seen for tips B10 and C10. However, most noteworthy was the difference in measured variance, which was considerably smaller in silicone compared to agar-agar. For silicone, the results show an increased radial-to-axial force ratio with increased tip angle.

On the right hand side of the figure, the data of the first loading cycle are compared to the data of subsequent cycles in silicone. A clear drop in the force ratio from the first to the second cycle was found for tips B10 and C10. For the other tip types, the first loading cycle may also exhibit a distinct response compared to subsequent cycles, but with a less clear effect.

### Force-displacement slopes

**Figure 6:**
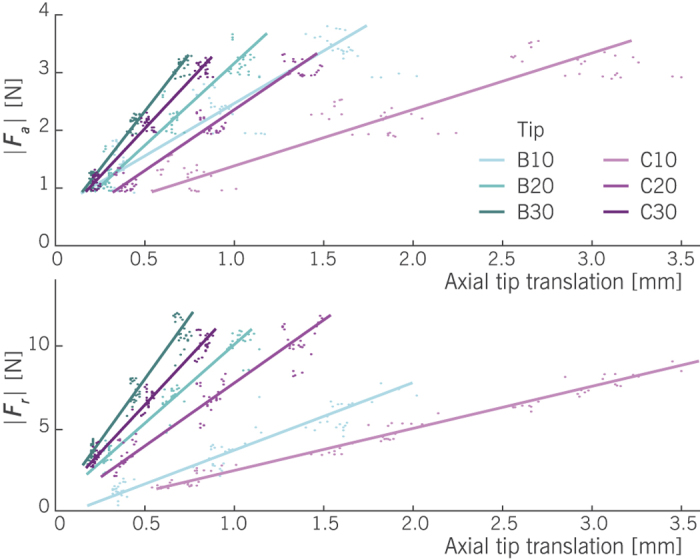
Axial force |*F*_*a*_| (top) and radial force |*F*_*r*_| (bottom) versus the axial insertion depth in silicone, using various needle tips. Linear least squares regression lines are also shown.

Figure 6 (top) shows the axial force as a function of the axial translation. Data fits in this figure are based on a least squares estimator of a linear regression model. These fits are used to model the slopes, i.e. effective stiffness estimates, of each of the studied tip types in silicone. The slopes are summarized in Table 1, along with the coefficients of determination of the linear fits. It can be seen that beveled tips had a larger slope than conical tips with a similar tip angle. The effective stiffness of tip B10 was 1.8 N/mm, compared to 1.0 N/mm for its paired conical tip version C10. However, the effect of tip angle in this study was larger than the effect of tip type (B/C). The effective stiffness values of pre-curved beveled and conical tips (B30 and C30) were 4.0 N/mm and 3.3 N/mm, respectively. The coefficients of determination of the linear models were all high, meaning that these models can largely explain the measured variability. We also looked at the residuals, which appeared randomly distributed around zero.

In a similar way, the radial force versus insertion depth is shown in Fig. 6 (bottom). Overall, the *F*_*r*_-slopes are higher than the *F*_*a*_-slopes, as is also shown in Table 1. Furthermore, bevel tips are more efficient than conical tips in the radial force buildup. For pre-curved bevel and conical tips (B30 and C30), the slopes were respectively 15.1 N/mm and 11.6 N/mm. The difference between radial and axial force components was presented in Fig. 5 by the radial-to-axial force ratio. This scaling factor largely explains the difference between the two graphs of Fig. 6. For instance, it can be seen that the *F*_*r*_-slopes for tips B10 and C10 are, on the whole, lower than their axial equivalents. The radial-to-axial force ratio is also smaller for these tip types.

## Discussion

The goal of this study was to quantify and compare tip-tissue contact forces for various tip shapes. It was assumed that the resultant force acting on the needle tip plays an important role in the direction and extent of needle steering, even though the needle shape is a result of loads acting along the full needle length. Current mechanics-based steering models often use a constant tip load, which relates to the tissue elasticity and rupture toughness^9^, with a fixed orientation, e.g. orthogonal to the bevel^4^. Evaluating the tip load for other tip shapes would allow for extensions of current models to other steering techniques and needle designs.

For needles that are embedded in tissue, it is not trivial to isolate the tip load from the force measured at the hub. To our awareness, only one study analyzed the load-distribution at the tip of beveled needles in detail^9^. The present work discusses both the magnitude and orientation of tip loads for various asymmetric tip shapes in a scaled experiment, i.e. by zooming in on the tip and directly attaching a force sensor to this region of interest. Tips with a 10 mm outer diameter were used to increase the tip-tissue contact surface, the force magnitudes, and the signal-to-noise ratio. A linear relation of the tip load with the frontal contact surface, and therefore a quadratic relation with diameter, was assumed. Deformations of the used tips during the insertions were considered to be negligible.

The experimental results showed that the accuracy of current navigation models may be improved by including a tip load vector with a variable orientation and size, as these factors were found to change with the insertion force. Under the assumption that a constant tip load orientation is required for a constant path curvature, this finding may explain why the path radii in some materials have been reported as constant, but not in others. Follow-up studies may result in analytical models for the tip load. These models may estimate bending forces more accurately for VR simulators, e.g. by means of an axial force and a tip load orientation estimate. In practice, axial force at the tip may, for instance, be measured by means of fiber Bragg gratings embedded in the needle.

When comparing the tip loads of beveled and conical tips, differences in the slopes of the force-displacement curves were seen. For both the insertion and bending force components, the slopes were consistently larger for beveled tips than for the matched conical tips. Although an efficient formation of bending forces is a relevant design criterion for the development of steerable needles, there are more factors to consider. An example is the desired degrees of freedom in device actuations. Conical-tip needles can be articulated to any direction to produce a geometrically similar shape, whereas beveled needles would have to rotate along their longitudinal axis to achieve the same.

An increased tip angle led to a quick increase of the force-displacement slopes. Most likely, the increased frontal surface area of these more pronounced asymmetric tips, contributed to this effect. This observation is in line with earlier studies that demonstrated increased path curvatures for pre-bent needle tips^11^. This stresses the potential benefit of tip articulations in needle steering. In addition, the dependence of the tip load orientation on the insertion force was found to be small for articulated tips. A near-constant force orientation would suggest a proportionality that can be easily implemented into current mechanics-based navigation models.

Repeated loading cycles are typically used for material pre-conditioning. Pre-conditioning is a common treatment in tissue characterization studies to ensure a repeatable reference state in the specimen structure^22^. For tip characterization studies, a repeatable tissue reference state is also important. In this study, a drop in the radial-to-axial force ratio was seen between the first and subsequent loading cycles. This effect appeared to vary per tip type and was the largest for tips B10 and C10. Silicone offered a more consistent force response than agar-agar. It allowed for repeated loading cycles per specimen, whereas agar-agar did not. In addition, the variability in force data during the first loading cycle was considerably lower in silicone than in agar-agar. Since it was noted during the Shore hardness tests that agar-agar easily deformed plastically, this effect may be the result of a difference in material elasticity. Elastic and plastic material deformations directly affect the force interactions at the tip. Besides the difference in variability among phantoms, this may explain the *F*_*r*_/*F*_*a*_ ratio effects for tips B10 and C10, see Fig. 5 (left), as these tips required the largest axial displacements.

Wang *et al*.^20^ showed that the mechanical characteristics of silicone-based specimens may be tailored with mineral oil to obtain stiffness and friction values with orders of magnitude comparable to tissue, whereas the effect on cutting force was found to be limited. This study motivated the choice for a silicone with a 40 wt.% paraffin-based oil. In terms of viscoelastic properties, hysteresis, and energy storage, silicone may exhibit a more elastic response than soft tissue^23^. Note that the main objective of the used simulant materials was not to resemble human tissue, but to provide a controllable and repeatable test environment to study tip loads. In this regard, the silicone phantom performed better than the 2.5 wt.% agar-agar phantom.

The research objective was to study tip-tissue interaction forces as an isolated part of the whole system. The whole system is more complex and other variables can interact with the discussed relations. Fracture phenomena, for instance, can affect the magnitude of the resultant tip force, including the bending force component. So can insertion velocity. For instance, velocity dependencies on the needle path were found for some tip types^8^, and not for others^24^. Insertion velocity can, in turn, affect the fracture phenomena^12^, complicating the set of acting relations. Whole-system mechanics may only be understood in a step-by-step approach. In the current work, fracture phenomena were not studied, and contact forces were measured at quasi-static states, i.e. after each tip translation. In a continuation of this work, these variables should be gradually introduced in order to converge to a whole-system representation of the tip-tissue mechanics.

One parameter that was not controlled in this study was the timing between loading steps and cycles. Tissue relaxation effects were visible when returning from *z*_3*N*_ to *z*_*ref*_. Typically, a tip was kept 30–60 s at each loading step. The presence of force residuals can be seen from Fig. 6, as the axial forces are not exactly at 1 N, 2 N, and 3 N. The increase in axial force between steps was, however, kept constant. For future studies it is suggested to implement an automated insertion process, with a sufficiently long and constant pause between the subsequent loading steps.

Finally, to arrive at explanatory needle steering models, the relations between the resultant tip-tissue load vector and other system variables, such as tip type, tissue type and insertion velocity, should be studied in detail. The current work analyzed the tip-tissue interaction forces of six different asymmetric needle tips in two phantom materials. This gave insight in the effect and value of using pre-bent, pre-curved, or actively articulated needle tips for steering applications. The increase in axial (*F*_*a*_-slope) and radial (*F*_*r*_-slope) forces with insertion depth were consistently higher for articulated bevel-tips than for conical tips with a similar tip angle. However, a stronger positive effect on these slopes was found for increased tip angles, e.g. by adding a pre-bend or pre-curve to the tip. For small tip angles, a vector orientation dependence on the applied axial load was observed. An increase of the insertion force caused the tip vector to orient more radially. In case a pre-bend or pre-curve was added to the tip, this dependence was reduced, and a nearly constant vector orientation was found. These findings relate to the correctness of presenting tip loads by a single, constant vector orientation, e.g. orthogonal to the bevel. This work is relevant for the formation of mechanics-based system models, for the optimization of tip designs, and for the proper actuation of shape-adaptable steerable needles.

## Additional Information

**How to cite this article**: van de Berg, N. J. *et al*. The influence of tip shape on bending force during needle insertion. *Sci. Rep.* **7**, 40477; doi: 10.1038/srep40477 (2017).

**Publisher's note:** Springer Nature remains neutral with regard to jurisdictional claims in published maps and institutional affiliations.

## Supplementary Material

Supplementary Figure S1

## Figures and Tables

**Table 1:** Summary of the linear least squares fits that describe the axial and radial force increase with insertion depth in silicone.

Tip	Slope *F*_*a*_ [N/mm]	*R*^2^	Slope *F*_*r*_ [N/mm]	*R*^2^
B10	1.8	0.79	4.1	0.91
B20	2.8	0.92	9.5	0.95
B30	4.0	0.98	15.1	0.94
C10	1.0	0.84	2.6	0.98
C20	2.1	0.94	7.6	0.92
C30	3.3	0.97	11.6	0.97

Presented are the slopes and the coefficients of determination (*R*^2^).
