# Supplementary material for: The influence of tip shape on bending force during needle insertion
Source: Sci Rep. 2017 Jan 11;7:40477. doi: 10.1038/srep40477 (PMC5225462; doi:10.1038/srep40477)
Supplement: Supplementary Figure S1 [file srep40477-s1.pdf]

The influence of tip shape on bending force during needle insertion

Nick J. van de Berg[1,*], Tonke L. de Jong[1], Dennis J. van Gerwen[1], Jenny Dankelman[1], and John J. van den Dobbelsteen[1]

[1]Delft University of Technology, BioMechanical Engineering, Delft, 2628CD, The Netherlands
[*]n.j.p.vandeberg@tudelft.nl

ABSTRACT

Steering of needles involves the planning and timely modifying of instrument-tissue force interactions to allow for controlled deflections during the insertion in tissue. In this work, the effect of tip shape on these forces was studied using 10 mm diameter needle tips. Six different tips were selected, including beveled and conical versions, with or without pre-bend or pre-curve. A six-degree-of-freedom force/torque sensor measured the loads during indentations in tissue simulants. The increased insertion (axial) and bending (radial) forces with insertion depth — the force-displacement slopes — were analyzed. Results showed that the ratio between radial and axial forces was not always proportional. This means that the tip load does not have a constant orientation, as is often assumed in mechanics-based steering models. For all tip types, the tip-load assumed a more radial orientation with increased axial load. This effect was larger for straight tips than for pre-bent or pre-curved tips. In addition, the force-displacement slopes were consistently higher for 1) increased tip angles, and for 2) beveled tips compared to conical tips. Needles with a bent or curved tip allow for an increased bending force and a decreased variability of the tip load vector orientation.

Supplementary information

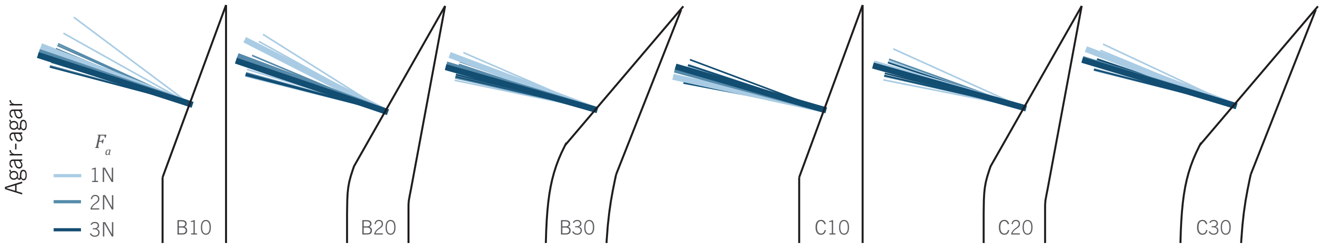

Figure S1. Summary of resultant force vector orientations per tip type and axial loading condition in agar-agar. The slightly thicker and longer lines present the median vector orientations per experimental condition. Note that agar-agar did not allow for repeated loading cycles. Since the vector orientation changed with repeated loading cycles in silicone, a direct comparison of this figure to Fig. 4 of the main document is not advised.
